# Simple transanal total mesorectal resection versus laparoscopic transabdominal total mesorectal resection for the treatment of low rectal cancer: a single-center retrospective case-control study

**DOI:** 10.3389/fsurg.2023.1171382

**Published:** 2023-07-27

**Authors:** Wei-Feng Yang, Wenbin Chen, Zijian He, Zixin Wu, Huilong Liu, Guanwei Li, Wang-Lin Li

**Affiliations:** School of Medicine, South China University of Technology, Guangzhou, China

**Keywords:** simple transanal total mesorectal excision, laparoscopic resection of low rectal cancer, low rectal cancer, sample quality, postoperative follow-up

## Abstract

**Aim:**

To evaluate the efficacy and safety of simple TaTNE in the treatment of low rectal cancer compared with laparoscopic transabdominal TME.

**Methods:**

We collected patients with low rectal cancer admitted to our hospital between January 2019 and November 2021 who received simple TaTME or laparoscopic transabdominal TME. The main outcome was the integrity of the TME specimen. Secondary outcomes were the number of lymph nodes dissected, intraoperative blood loss, operative time, surgical conversion rate, Specimen resection length, circumferential margin (CRM), and distal resection margin (DRM), complication rate. In addition, the Wexner score and LARS score of fecal incontinence were performed in postoperative follow-up.

**Results:**

Pathological tissues were successfully resected in all patients. all circumferential margins of the specimen were negative. Specimen resection length was not statistically significant (9.94 ± 2.85 vs. 8.90 ± 2.49, *P* > 0.05). The incidence of postoperative complications in group A (*n* = 0) was significantly lower than that in group B (*n* = 3) (*P* > 0.05). There was no significant difference in operation time between group A and group B (296 ± 60.36 vs. 305 ± 58.28, *P* > 0.05). Among the patients with follow-up time less than 1 year, there was no significant difference in Wexner score and LARS score between group A and group B (*P* > 0.05). However, in patients who were followed up for more than 1 year, the Wexner score in group A (9.25 ± 2.73) was significantly lower than that in group B (17.36 ± 10.95) and was statistically significant (*P* < 0.05).

**Conclusion:**

For radical resection of low rectal cancer, Simple TaTME resection may be as safe and effective as laparoscopic transabdominal TME, and the long-term prognosis may be better.

## Highlights

• Simple TaTME was innovated by our team.
• Simple TaTME may also be safe and effective compared to laparoscopic transabdominal TME.
• From the analysis of follow-up results, the recovery of anal function after TaTME alone may be better.

## Introduction

Rectal cancer, one of the most common malignant tumors of the gastrointestinal tract, has risen substantially incidence and mortality year by year. At present, the incidence of colorectal cancer is the third highest in the world (1). Although the multidisciplinary treatment of rectal cancer is the standard of care in clinical practice, surgery remains the primary curative modality (2). More importantly, the total mesorectal excision (TME) has become the gold standard for surgical treatment of rectal cancer (3). And with the recent progression of minimally invasive techniques for colorectal cancer, laparoscopic transabdominal TME has become one of the main surgical methods for the treatment of rectal cancer (4). Previous studies have established that laparoscopic treatment of rectal cancer has more advantages than laparotomy in terms of short-term efficacy and long-term efficacy (5). But it has significant shortcomings in the treatment of ultra-low rectal cancer patients with obesity, pelvic stenosis, and male prostatic hypertrophy (6). Transanal total mesorectal excision (TaTME) includes features of the TME principle and the natural orifice specimen extraction (NOSES) concept, providing a new evidence-based treatment option (7, 8). In 2010, transanal TME (TaTME) surgery in the field of rectal surgery was first reported by Sylla et al. in the United States (9). Although it raises controversy in clinical application, some scholars believe that this procedure is a relatively safe and effective supplementary procedure, especially for patients with low rectal cancer (10, 11). On the basis of TaTME, our team innovatively proposed a simple surgical approach for TaTME, using conventional multiport laparoscopy instead of single-port laparoscopy, and transanal resection of cancer tissue under direct vision, making the operation simpler and easier. Eight patients who underwent simple TaTME in the early stage were followed up, and it was found that the patients recovered quickly after operation, the quality of the specimen was high, and the postoperative anal function was good. In addition, surgery has a negative impact on the patient's overall health. Reasonable preparation before operation will affect the postoperative recovery of patients. Some studies have found that mechanical bowel preparation combined with oral antibiotic bowel preparation can reduce the incidence of postoperative infection, but does not affect the mortality (12). And some preoperative rehabilitation interventions also have certain effects, such as preoperative exercise training, nutritional support, psychological mediation, which can enhance patients' adaptability and improve patients' postoperative quality of life (13). Therefore, this study will include patients who received the same preoperative preparation. Our study will collect patients with low rectal cancer who underwent simple TaTME surgery or laparoscopic transabdominal TME surgery from January 2019 to November 2021 in our hospital for a retrospective case-control study. This study will compare the therapeutic effects of the two surgical methods to provide a reliable basis for the study of surgical treatment of low rectal cancer.

## Materials and methods

### Patient selection

Patients with low rectal cancer treated at hospital from January 2019 to November 2021 were retrospectively analyzed. These patients underwent colonoscopy, pelvic magnetic resonance imaging (MRI) and/or endorectal ultrasonography, thoracoabdominal computed tomography (CT) scans, and histopathological examination preoperatively. They were all diagnosed with low rectal cancer (tumor no more than 5 cm from anal verge). Patients included in this study were divided into group A and group B according to the surgical methods received, in which patients who underwent simple TaTME surgery were in group A and patients who underwent laparoscopic transabdominal TME surgery were in group B.

The inclusion criteria were as follows: (1) Patients with histologically confirmed moderately and poorly differentiated rectal adenocarcinoma; (2) The tumor margin was no more than 5 cm from the anal verge; (3) Patients without distant metastasis after preoperative evaluation; The exclusion criteria were as follows: (1) Patients with recurrent or tumor invading the external anal sphincter; (2) Patients with colorectal cancer with vital organ dysfunction; (3) Patients with acute intestinal obstruction or perforation. All cases in this study were approved by the institutional review board of our hospital medical center.

### Preoperative adjuvant therapy

Patients were staged before surgery by pelvic magnetic resonance imaging (MRI) and/or intrarectal ultrasound, chest and abdominal computed tomography (CT), and histopathology. Patients who met the criteria of “Chinese Clinical Guidelines for Colorectal Cancer” received neoadjuvant chemoradiotherapy. Patients receiving neoadjuvant chemoradiotherapy undergo surgery 8–12 weeks after completion of adjuvant therapy.

### Surgical procedures

All operations were performed by our senior colorectal surgery team, and the operators underwent regular endoscopic training and learning curve. Routine mechanical bowel preparation was performed actively before surgery. After successful anesthesia, the patient was placed in the modified lithotomy decubitus position and routinely disinfected and draped.

### Simple TaTME procedure

First step transanal procedure. After perianal disinfection, the rectum was fully exposed with a colonic retractor, and an electroknife incision was made in the lumen 1–2 cm below the tumor under direct vision. Secondly, it was closed with pouch suture, and then the whole layer circumferential cleaning was performed. The transanal anatomical separation of the rectum is then performed under direct vision, rather than using a gloved single-hole platform and constructing an inflatable pelvis. We'll start by dissecting the back side. Then we cut the anterior side, cut the anterior longitudinal muscle, and then separate it forward and down. Lateral stripping is performed after anterior and posterior separation. The anatomy extends beyond the upper margin of the tumor or above the anorectal ring.

Second step transabdominal procedure. In order to simplify the surgical process, we used traditional multiport laparoscopy instead of single-port laparoscopy. A medial and lateral approach was used to separate the nodes using an ultrasound knife and absorbable clamp, while regional lymph nodes were dissected. We then dissected the upper part of the rectum until the tumor merged with the previous transanal surgical plane.

Finally, we removed specimens via the anus, performed an end-to-end coloanal anastomosis using a stapler, and created a prophylactic ileostomy to ensure better healing of the anastomosis (Supplementary Figure S1, Supplementary Videos S1, S2).

### Laparoscopic transabdominal TME

First, laparoscopy was routinely placed, and the specimen was freed to more than 1–2 cm from the distal rectum and divided using a cutter. Then, a 5 cm oblique incision was made in the left lower quadrant and the bowel tube was divided to 10 cm above proximal tumor. Next, the mesentery was divided and the diseased intestinal segment was removed from the left lower quadrant incision. Finally, an end-to-end anastomosis was performed with a stapler and an artificial ileostomy was performed in the right lower quadrant.

All the above surgical operations strictly follow the basic principles of surgical operation and the principles of TME.

### Assessment measures

Assessment of specimens: Postoperative pathological stage, circumferential resection margin analysis of specimens, number of dissected lymph nodes, and sample length; Intraoperative and postoperative related indicators: operation time, defecation time and whether to convert to laparotomy; Post-operative complications: Postoperative wound infection, anastomotic leakage, anastomotic stenosis; Postoperative follow-up indicators: Recurrence, follow-up time, low anterior resection syndrome (LARS) score and postoperative Wexner score. Postoperative Wexner score assesses postoperative anal function in patients, with higher scores indicating poor function.

### Statistical analysis

Data analysis was performed using SPSS 25.0 software. Measurement data were presented as mean ± standard deviation. Independent sample *t*-test was used for comparisons between groups. Enumeration data were expressed as percentages and *χ*^2^ test was used. *P* < 0.05 was considered statistically significant.

## Results

### Clinical characteristics of patients

Given that the simple TaTME surgery proposed by our team was first performed in our hospital in 2019. In this study, we collected patients who received laparoscopic transabdominal TME surgery during the same period as the control. From 2019 to 2021, a total of 171 patients received surgical treatment for rectal cancer in our hospital, including 55 patients whose rectal cancer was less than 5 cm away from the anal margin. Four patients were excluded due to inadequate clinical data. Finally, a total of 51 patients were enrolled in the study, including 17 patients who underwent simple TaTME surgery and 34 patients who underwent laparoscopic transabdominal TME surgery.

The basic characteristics compared between the two groups included gender, age, body mass index (BMI), preoperative chemoradiotherapy, TNM stage of the tumor, underlying diseases, and history of abdominal surgery. There were no statistically differences in gender, age, preoperative chemoradiotherapy, and TNM stage of the tumor between the two groups. Compared with group A, patients with underlying diseases and history of abdominal surgery accounted for a higher proportion in group B. More importantly, the BMI value of group B was lower than that of group A, and the difference was statistically significant (*P* < 0.05). In addition, a total of 22 patients completed the protocol, 6 in group A and 16 in group B. Surgery was scheduled for weeks 8–12 after neoadjuvant radiotherapy (see Table 1).

**Table 1:** Clinical characteristics of patients with low rectal cancer *n* (%).

Variable	Group A, *n* = 17	Group B, *n* = 34	*P* value (*P* < 0.05)
Sex			0.546
Male	11 (64.71)	19 (55.88)	
Female	6 (35.29)	15 (44.12)	
Age^a^	62.88 ± 10.37	63.74 ± 11.07	0.774
BMI^a^	23.54 ± 2.89	21.40 ± 3.34	0.024*
Neoadjuvant radiotherapy	6 (35.29)	16 (47.06)	
Adjuvant radiotherapy	0 (0)	0 (0)	
Adjuvant chemotherapy	8 (47.06)	9 (26.47)	
TNM stage			0.700
Stage Ⅰ	4 (23.53)	5 (14.71)	
Stage Ⅱ	7 (41.18)	14 (41.18)	
Stage Ⅲ	6 (35.29)	15 (44.12)	
Underlying disease			
Hypertension	0 (0)	9 (26.47)	
Diabetes	1 (5.88)	4 (11.76)	
Previous abdominal operation	0 (0)	6 (17.65)	

BMI, Body mass index; TNM, Tumor node metastasis.

^a^ Values are mean ± SD. Group A, A modified approach with simplified transanal total mesorectal excision; Group B, A classical approach with transabdominal resection of low rectal cancer.

*P < 0.05.

### Surgical results

In this study, we assessed the operative procedure time, the time of postoperative hospital stay, time to first defecation after surgery, and relevant measures for tumor resection assessment for both procedures. The operative time in group A (296 ± 60.36 min) was shorter than that in group B (305 ± 58.28 min; *P* = 0.615), But the difference was not significant. Similarly, the time of postoperative hospital stay and first postoperative defecation time were also not significantly different between group A and group B. In addition, there was no significant difference between the two groups in the distance from the lower edge of the tumor to the anal verge, the length of bowel resection, the differentiation of the tumor tissue, the circumferential resection margin, and lymph node dissection (see Table 2).

**Table 2:** Clinicopathological outcomes *n* (%).

Variable	Group A, *n* = 17	Group B, *n* = 34	*P* value (*P* < 0.05)
Total operating time^a^	296 ± 60.36	305 ± 58.28	0.615
Blood loss^a^	217.65 ± 157.06	160.88 ± 94.21	0.113
Hospital stays after operation^a^	9.41 ± 3.34	9.68 ± 4.45	0.813
Distance of tumors from distal margin^a^	4.03 ± 0.86	4.32 ± 0.75	0.251
Tumor diameter^a^	4.07 ± 1.56	3.12 ± 1.29	0.039*
Length of specimen^a^	9.94 ± 2.85	8.90 ± 2.49	0.209
Tumor differentiation			
Poorly differentiated	1 (5.88)	2 (5.88)	
Moderately differentiated	16 (94.12)	32 (94.12)	
Circumferential resection margin			
Positive	0 (0)	0 (0)	
Negative	17 (100)	34 (100)	
Lymph nodes harvested	13.35 ± 7.72	14.09 ± 7.69	0.75
Bowel movement (days)	3.76 ± 2.11	3.50 ± 2.91	0.713

^a^ Values are mean ± SD. Group A: A modified approach with simplified transanal total mesorectal excision; Group B: A classical approach with transabdominal resection of low rectal cancer.

*P < 0.05.

### Postoperative complications of surgery

No postoperative complications occurred in group A, but 3 patients in group B had different complications. Among them, 1 patient had postoperative wound infection and 2 patients had anastomotic leakage. None of the 3 cases had a second operation and recovered with clinical care. The difference between the two groups was not statistically significant (see Table 3).

**Table 3:** Postoperative complications in patients with low rectal cancer *n* (%).

Variable	Group A, *n* = 17	Group B, *n* = 34	*P* value (*P* < 0.05)
Anastomotic leakage	0 (0)	2 (5.88)	
Anastomotic stricture	0 (0)	0 (0)	
Postoperative inflammatory intestinal obstruction	0 (0)	0 (0)	
Wound infection	0 (0)	1 (2.94)	
Reoperation	0 (0)	0 (0)	
Postoperative complications	0 (0)	3 (8.82)	0.207

Data are presented as *n* (%). Group A, A modified approach with simplified transanal total mesorectal excision; Group B, A classical approach with transabdominal resection of low rectal cancer.

### Follow-up results

The follow-up results showed that 17 patients were followed up in group A, 1 relapsed and 1 died (due to underlying disease); 34 patients were followed up in group B, 4 relapsed (including 1 death) and 3 died (1 metastasis and 2 due to underlying disease). The recurrence rate was 5.88% in group A and 11.76% in group B. There was no significant difference between the recurrence rate of group A and the recurrence rate of group B (*p* > 0.05).

In addition, the postoperative follow-up of the two groups was analyzed according to whether the follow-up time exceeded 1 year. Among the cases with follow-up time less than 1 year, the average follow-up time was 8.91 ± 2.41 months in 5 cases of group A and 8.05 ± 2.25 months in 9 cases of group B. The Wexner score (19.00 ± 10.92) and LARS score (9.00 ± 2.16) in group A were lower than those in group B (30.38 ± 8.57) and LARS score (12.13 ± 4.02), but there was no significant difference between the two groups. Among the patients who were followed up for more than1 year, the mean follow-up time was 28.22 ± 5.67 months in group A and 27.67 ± 8.71 months in group B. The LARS score of group A (20.50 ± 11.09) was lower than that of group B (19.68 ± 10.76), and there was no significant difference in the LARS score between the two groups, but the Wexner score of group A (9.25 ± 2.73) was significantly lower than that of group B (17.36 ± 10.95), and the difference was statistically significant (*P* < 0.05) (see Tables 4–6).

**Table 4:** Postoperative follow-up results of patients with low rectal cancer *n* (%).

Variable	Group A, *n* = 17	Group B, *n* = 34	*P* value (*P* < 0.05)
Follow-up (cases)	17 (100)	34 (100)	
Recurrence	1 (5.88)	3 (8.82)	0.693
Death	1 (5.88)	3 (8.82)	0.715

Data are presented as *n* (%). Group A, A modified approach with simplified transanal total mesorectal excision; Group B, A classical approach with transabdominal resection of low rectal cancer.

**Table 5:** Postoperative follow-up results of patients with low rectal cancer *n* (%) (follow-up < 1years).

Variable	Group A, *n* = 17	Group B, *n* = 34	*P* value (*P* < 0.05)
Follow-up < 1 years (cases)	5	9	
Follow-up < 1 years (months)	8.91 ± 2.41	8.05 ± 2.25	0.534
Wexner Score	19.00 ± 10.92	30.38 ± 8.57	0.075
LARS Score	9.00 ± 2.16	12.13 ± 4.02	0.111

Values are mean ± SD. Group A, A modified approach with simplified transanal total mesorectal excision; Group B, A classical approach with transabdominal resection of low rectal cancer.

**Table 6:** Postoperative follow-up results of patients with low rectal cancer *n* (%) (follow-up > 1years).

Variable	Group A, *n* = 17	Group B, *n* = 34	*P* value (*P* < 0.05)
Follow-up > 1 years (cases)	12	25	
Follow-up > 1 years (months)	28.22 ± 5.67	27.67 ± 8.71	0.843
Wexner Score	9.25 ± 2.73	17.36 ± 10.95	0.020*
LARS Score	20.50 ± 11.09	19.68 ± 10.76	0.796

Values are mean ± SD. Group A, A modified approach with simplified transanal total mesorectal excision; Group B, A classical approach with transabdominal resection of low rectal cancer.

*P < 0.05.

## Discussion

Classical laparoscopic transabdominal TME can remove both tumor tissue and mesentery, blood vessels, lymphoid tissue, and adipose tissue that match the tumor, greatly reducing the possibility of local recurrence (14). It has the advantages of clear surgical field, easy operation, rapid postoperative recovery, and few complications (4). However, how to remove low rectal cancer tissue with high quality in the narrow pelvic space faces challenges. The proposal of transanal total mesorectal excision fills the shortcomings in the clinical application of laparoscopic total mesorectal excision. Its unique “downward and upward” anatomical angle avoids the “chopstick effect” resulting from space narrowing during laparoscopic surgery, particularly benign prostatic hyperplasia, pelvic narrowing, visceral fat, and/or BMI > 30 kg/m2 (15). As a current research hotspot in the field of colorectal and anal surgery, the procedure has been applied in clinical practice in many countries and a number of randomized controlled studies have been carried out (16–18), but variability in reported findings still precludes firm conclusions. Some studies found that TaTME was a procedure that was not inferior to laparoscopic surgery. TaTME can also achieve clinical effect comparable to laparoscopic TME. More importantly, evidence suggests that TaTME is also safe and effective compared to laparoscopic TME (19, 20). However a Norwegian national study came to the opposite conclusion so that TaTME was halted nationwide (21). At the same time, with the clinical application, the optimal surgical platform and surgical methods of TaTME surgery are also continuously innovating. For example, ChangXu et al. used a modified TaTME method with simple customized instruments to achieve high-quality TME for the treatment of male low rectal cancer (22).

Based on these considerations, our retrospective study found that the two types of surgery were similar in sample quality, postoperative complications, operative time, and surgical bleeding, and postoperative follow-up showed that the recovery of anal function in group A was better than that in group B. Therefore, we believe that simple TaTME may achieve similar therapeutic effect as laparoscopic transabdominal TME in the treatment of low rectal cancer, but we need to collect more cases for further confirmation in the future.

The comparison of basic characteristics between the two groups showed that the two groups showed similar performance in terms of gender ratio, age distribution, preoperative adjuvant therapy, presence of underlying diseases (including diabetes, hypertension, etc.) and history of abdominal surgery. Remarkably, BMI mean were 23.54 ± 2.89 in group A and 21.40 ± 3.34 in group B, and the difference was statistically significant (*P* < 0.05). However, mean BMI values were within the normal range in both groups. Therefore, it is not clear that group A is superior to group B in the treatment of obese patients, although existing studies have shown the advantage of transanal mesenterectomy in the treatment of obese patients (21).

The resection margin, circumferential resection margin (CRM), and integrity of the mesorectum at the distal end of the tumor are the three core indicators for the quality assessment of total mesorectal excision specimens, while they are closely related to the patient's postoperative recovery status (23). Theoretically, Simple TaTME can obtain high-quality case specimens because of improved surgical operating space and surgical field, and the existing clinical findings do (24, 25). In this study, the resection margin and CRM were negative. From the analysis of postoperative pathological tissue, there were no significant differences between the two groups in the frequency of regional lymph node dissection, the length of specimen resection, and pathological stages. Thus, simple TaTME may obtain specimens of the same quality as laparoscopic transabdominal TME.

The incidence of postoperative complications is one of the important indicators reflecting the safety of surgery. Postoperative complications of low rectal cancer resection mainly include anastomotic leakage, anastomotic stricture, postoperative inflammatory intestinal obstruction, and incision infection. Among them, anastomotic leakage occurs most frequently. The mild cases will have fever, pelvic infection, anastomotic stricture and other complications. The severe cases will lead to permanent stoma or severe infection or even life-threatening. A large number of studies have now shown that transanal TME surgery does not lead to a higher complication rate (26). The procedure may be considered safe. And a mate analysis involving 899 patients show no difference in complication rates between the two procedures (27). The same holds true for our findings. Our results showed that no postoperative complications occur in group A, and 3 patients in group B has different complications, indicating that simple TaTME may be safe for patients with low rectal cancer. However, a multicenter study by the European Society of Coloproctology (ESCP) showed that the incidence of anastomotic stoma after transanal total mesorectal excision (12.9%) is higher than that after non-transanal laparoscopic total mesorectal excision (8.9%), especially for male patients (28). These controversies may be caused by the complexity of transanal TME surgical procedures and can be resolved by special training.

Patient recovery after surgery is an important part of surgical evaluation. From the follow-up results of this study, the recurrence rate was 5.56% in group A and 11.76% in group B. The recurrence rate was significantly lower in group A. In cases with a follow-up time of less than 1 year, the short-term postoperative recovery of anal function was comparable between the two procedures, and the same conclusion has been reached in previous studies (29). Conversely, anal function recovery was better in group A compared to group B in cases with follow-up longer than 1 year. Thus, both surgical methods will have different degrees of low anterior resection syndrome and anal incontinence after surgery, but with the extension of time, the recovery of anal function in group A is better than that in group B. However, in a single-center retrospective study comparing transanal and transabdominal total mesorectal excision for the occurrence of postoperative low anterior resection syndrome (LARS), the authors conclude that both procedures had similar recovery of bowel function in the long run (30). Even transanal total mesorectal excision has been shown to be associated with worse postoperative bowel dysfunction (31). Limited research evidence is not available to draw firm conclusions on this issue, and studies with larger sample sizes and longer follow-up times are needed.

In addition, the evaluation of surgical process is also an important indicator, mainly reflected in the operation time. Because of the advantages of bidirectional operation in TaTME surgery, the operation time should be significantly less than laparoscopic transabdominal TME surgery. However, there was no obvious difference in operation time between the two procedures (A time = 296 ± 60.36 min; B time = 305 ± 58.28 min) in our study. Due to objective conditions, surgeons use a single-team stepwise approach rather than a two-team simultaneous abdominal and anal approach in the simple TaTME procedure, which means that the operating platform needs to be replaced during surgery. thereby prolonging the operation time and resulting in comparable time between the two procedures. Reports have demonstrated that transanal TME performed by both teams significantly reduced operative time (32). A study of 34 patients who underwent transanal TME resulted in similar conclusions (33).

In this study, we also recognize some limitations: it was a retrospective study with a small number of included cases and a short follow-up period. Future studies on simple TaTME require larger prospective studies and longer postoperative follow-up.

## Conclusion

The results of this study suggest that simple TME surgery may have similar tumor and functional outcomes as laparoscopic transabdominal TME surgery, and anal functional recovery may be better in the long run.

## Data Availability

The original contributions presented in the study are included in the article/**Supplementary Material**, further inquiries can be directed to the corresponding author.
