# Supplementary material for: Simple transanal total mesorectal resection versus laparoscopic transabdominal total mesorectal resection for the treatment of low rectal cancer: a single-center retrospective case-control study
Source: Front Surg. 2023 Jul 27;10:1171382. doi: 10.3389/fsurg.2023.1171382 (PMC10413134; doi:10.3389/fsurg.2023.1171382)
Supplement: Supplementary file 5 [file Datasheet1.pdf]

Supplementary materials:

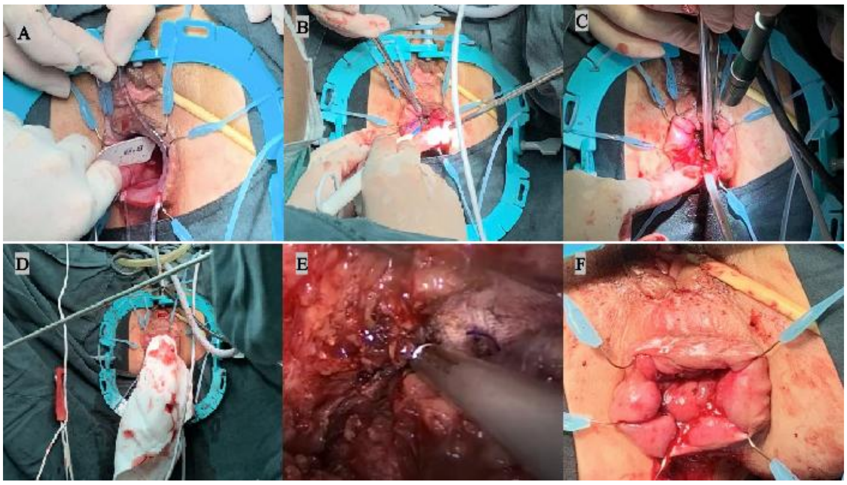

Figure 1. Simple TaTME operation procedure. A. Dilate the anus and determine the location of the lesion; B. Incision was made 1-2cm below the tumor under direct vision; C. Transanal separation of pathological tissue; D, after the completion of the transanal operation, use gauze to plug the anus to avoid air leakage; E. Transabdominal laparoscopy was used to separate pathological tissue until it merged with the transanal operating plane; F. Anal appearance after completion of surgical procedure.
